# Specific techniques for right sleeve lower lobectomy: four case reports

**DOI:** 10.1186/s40792-021-01123-9

**Published:** 2021-02-03

**Authors:** Hirokazu Hamasaki, Chika Shirakami, Tatsuya Yamada, Yamato Motooka, Kosuke Fujino, Koei Ikeda, Makoto Suzuki

**Affiliations:** Department of Thoracic Surgery, Kumamoto University Hospital, 1-1-1 Honjo, Chuo-ku, Kumamoto, 860-8556 Japan

**Keywords:** Lung cancer, Right sleeve lower lobectomy, Pericardiotomy, Interlobar dissection

## Abstract

**Background:**

Right sleeve lower lobectomy is rarely performed because pulmonary function of the middle lobe is not spared to the extent of the other lobes and achieving a proper bronchial anastomosis is technically more difficult than other sleeve lobectomies.

**Case presentation:**

We performed four right sleeve lower lobectomies and had good clinical outcomes using specific technical options, such as telescope anastomosing, pericardiotomy, interlobar dissection between the upper and middle lobes, and angioplasty of the lower pulmonary artery, if needed.

**Conclusions:**

The cases presented herein demonstrated that a right sleeve lower lobectomy is one option by which to preserve the middle lobe using specific techniques and is thus recommended in select patients.

## Background

Sleeve lobectomy is a common surgical method that requires technical skill to avoid bi-lobectomy or pneumonectomy and to preserve pulmonary function in patients with lung cancer [1–3]. A right sleeve lower lobectomy is an infrequently used option, thus right lower and middle lobectomies are usually performed because pulmonary function of the middle lobe is preserved to a lesser extent than the other lobes. In addition, achieving a proper anastomosis is technically difficult because of the differences in bronchial caliber and the anastomotic tension is much greater than in other sleeve lobectomies. Herein we report four patients who underwent right sleeve lower lobectomies with specific technical options.

## Case presentation

We retrospectively studied four patients who underwent right sleeve lower lobectomies for primary non-small cell or metastatic lung tumor from 2015–2018 in the Department of Thoracic Surgery (Kumamoto University Hospital). The clinical characteristics of the patients are shown in Table 1. All patients had tumors in the right S6 without clinical lymph node involvement. No patient received neoadjuvant treatment before surgery.

**Table 1:** The clinical data and the operative procedures

Case	Gender	Age	Histology	cTNM	c-stage	Suture of the bronchus	Management of		Pericardiotomy	Interlobar dissection between	Angioplasty of the pulmonary
							different calibers			the upper and middle lobes	artery of the middle lobe
1	Male	75	Adenocarcinoma	T1cN0M0	IA3	Interrupted	Telescope		+	−	−
2	Male	73	Squamous cell carcinoma	T2bN0M0	IIA	Interrupted	Telescope		−	+	+
3	Female	61	Atypical carcinoid	T1cN0M0	IA3	Interrupted and continuous suture	Telescope and sewing		+	+	−
4	Female	68	Metastatic adenocarcinoma from colon cancer			Interrupted	Telescope		+	+	−

The operative procedures are shown in Table 1. Specifically, a posterolateral thoracotomy was performed via the fifth intercostal space in all patients. The truncus intermedius was cut close to the second carina, while the middle lobe bronchi were cut at the orifice of the middle lobe bronchus. Thus, caliber disparities were large and distant for each bronchus. All bronchial stumps were negative for malignancy based on intraoperative pathologic examinations.

**Fig. 1:**
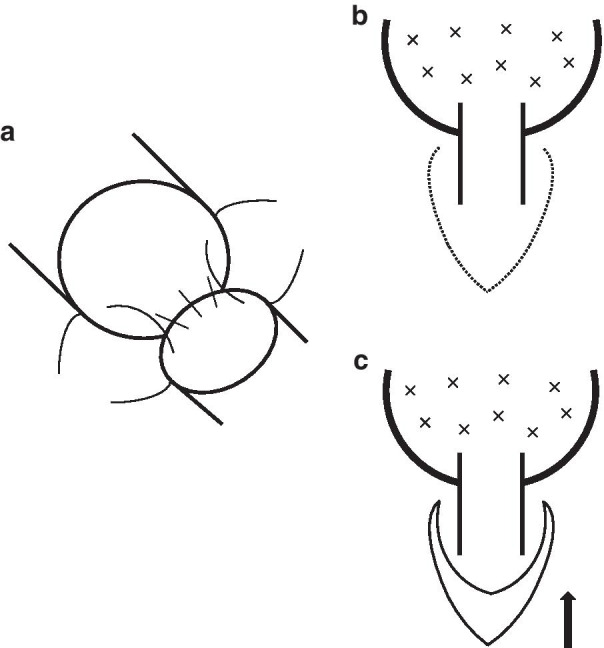
Intraoperative scheme. **a** Telescope anastomoses were performed. **b** Pericardial incision line (dotted line) of the pericardiotomy at the lower edge of the superior pulmonary vein. **c** The middle lobe was passively moved approximately 1.5 cm toward the cranial side (black arrow) after the pericardiotomy

To correct caliber disparities, telescope anastomoses were performed in all cases (Fig. 1a); a sewing membrane site of the truncus intermedius was added in one case. End-to-end anastomoses were sutured using 4-0 PDS (Ethicon) interrupted sutures in three cases, interrupted sutures for the cartilaginous portion, and a continuous suture for the membranous portion in one case.

**Fig. 2:**
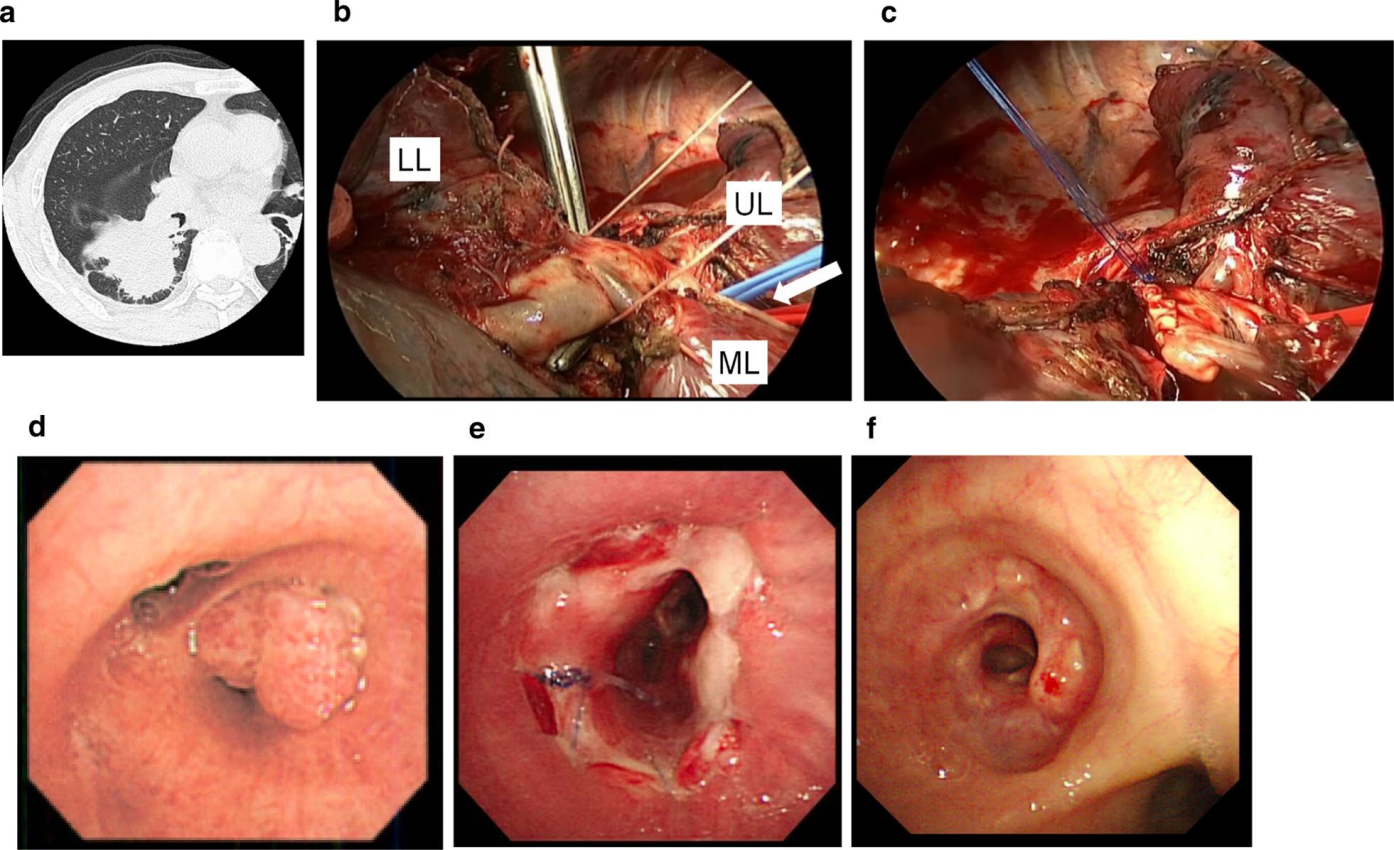
Images of a representative case (case 2). **a** Computed tomography image before surgery. **b** Intraoperative photograph. Because the tumor invaded the lower pulmonary artery, the pulmonary branch to the middle lobe was not preserved by an auto-suturing device or simple ligation. Interlobar dissection between the upper and middle lobes had already been performed; white box arrow. UL: upper lobe, ML: middle lobe, LL: lower lobe. **c** Angioplasty of the pulmonary artery was performed. Bronchoscopic findings. **d** Before surgery. The tumor occluded the orifice of the lower bronchus. **e** One week after surgery. The stump of the middle lobe bronchus was completely within the truncus intermedius. **f** Six months after surgery. The anastomotic site was covered with clean mucosa and the patency was good

To release the tension of the anastomoses, a pericardial incision at the inferior margin of the superior pulmonary vein was performed in three cases, but not case 2. As a result, the middle lobe was passively placed approximately 1.5 cm from the cranial side (Fig. 1b, c). Also, in addition to the above procedure, to release and align the bronchial axes, an interlobar dissection between the upper and middle lobes was performed in three cases, with the exception of case 1. The anastomoses were covered with pericardial fat tissue with stems in all cases. Due to tumor involvement in the lower pulmonary artery and to preserve blood flow in the middle lobe, angioplasty of the pulmonary artery was performed in case 2. Images of a representative case (case 2) are shown in Fig. 2.

There was no pathologic lymph node involvement in this series (Table 2). Bronchial anastomotic leakage was present in case 1, who did not undergo interlobar dissection between the upper and middle lobes. The patient received chest re-tube drainage and antibiotics, and the fistula was completely resolved 3 weeks after therapy. The postoperative courses were uneventful in the other cases. No patient needed oxygen therapy at the time of hospital discharge. Case 4, who had lung metastases from colon cancer, had multiple pulmonary metastases 18 months after the sleeve lobectomy, but all patients were alive during the observation period (Table 2).

**Table 2:** Pre- and post-pulmonary functions and clinical outcomes

Case	Lymph node	Preoperative	Preoperative	Postoperative	Postoperative	Anastomotic leakage	Prognosis
	metastasis	FEV1.0(L)	FEV1.0/BSA^a^ (L/m^2^)	FEV1.0 (L)	FEV1.0/BSA (L/m^2^)		
1	0/19	1.55	1.01	1.24	0.86	+	3y2m alive without tumor
2	0/36	2.01	1.46	1.62	1.14	−	1y9m alive without tumor
3	0/21	1.98	1.17	1.9	1.10	−	3y1m alive without tumor
4	0/6	2.00	1.35	ND^b^	ND	−	2y10m alive with tumor, multiple lung metastasis

^a^BSA; body surface area (L/m2)

^b^ND; not done

## Discussion

When the tumor infiltrates the orifice of the right lower bronchus, bi-lobectomy of the lower and middle lobes or lower sleeve lobectomy are the procedures of choice for lung cancer arising from the right lower lobe. Although sleeve lobectomy is a useful and common alternative for preserving pulmonary function, a right lower sleeve lobectomy is rarely performed due to the large caliber difference and strong tension on the bronchial anastomosis. Therefore, bi-lobectomy of the lower and middle lobes is likely to be selected rather than right lower sleeve lobectomy. However, because postoperative function and quality of life will be maintained better than bi-lobectomy of the lower and middle lobes, we selected right lower sleeve lobectomy using the above-mentioned technical procedures and had good clinical courses in our series.

There are few reports pertaining to right sleeve lower lobectomy [4–6]. Ohata et al. [4] recommended a bronchial flap to correct a bronchus caliber disparity [4]. We selected telescope anastomosis because we cut the orifice of the middle lobe bronchus and truncus intermedius close to the second carina to assure that the cut margins of each bronchus were negative for malignancy. Inevitably, the bronchus caliber disparity was large, thus a telescope anastomosis was performed with modifications to release the tension, as described below. Although the technical difficulty of suturing the anastomosis may increase, connecting short bronchial stumps may have been advantageous in terms of angiogenesis at the anastomotic site. In case 3, the caliber difference was excessive, so that the membranous part of the truncus intermedius site was sewn and anastomosed telescopically.

To release the anastomosis tension, we used several well-known techniques used in sleeve resection, such as dissection of the pulmonary ligament or pericardiotomy of the inferior pulmonary vein for sleeve upper lobectomy [7–9]. In our series, a pericardiotomy in the lower half of the superior pulmonary vein increased the mobility of the middle lobe, resulting in release of the bronchial anastomosis tension. Also, we emphasize the importance of interlobar dissection between the upper and middle lobes [4] and angioplasty of the lower pulmonary artery to preserve the blood flow to the middle lobe. Interlobar dissection between the upper and middle lobes may be essential to align the bronchial axes at the anastomosis, otherwise unilateral tension in the bronchial anastomosis persists when the upper and middle lobes swell, possibly resulting in suture failure. A bronchial anastomotic fistula developed in case 1. We did not perform interlobar dissection between the upper and middle lobes. Also, because bronchial artery preservation to the middle lobe cannot be expected during sleeve lobectomy and decreased blood flow may cause bronchial anastomotic insufficiency or anastomotic stenosis, the arteriovenous blood flow preservation to the middle lobe is essential.

By preserving the middle lobe, the postoperative pulmonary function was maintained and the quality of life was good without oxygen inhalation. It has been reported that pulmonary function after sleeve lobectomy is maintained as much as that following a lobectomy [3].

## Conclusion

In conclusion, a right sleeve lower lobectomy is one option by which to preserve the middle lobe using specific techniques and is recommended in select patients.

## Data Availability

Not applicable.

## References

[1] Melloula E, Eggerb B, Kruegera T, Chenga C, Mithieuxa F, Ruffieuxc C (2008). Mortality, complications and loss of pulmonary function after pneumonectomy vs. sleeve lobectomy in patients younger and older than 70 years. Interact Cardiovasc Thorac Surg..

[2] Berthet JP, Paradela M, Jimenez MJ, Molins L, Gomez-Caro A (2013). Extended sleeve lobectomy: one more step toward avoiding pneumonectomy in centrally located lung cancer. Ann Thorac Surg.

[3] D’Andrilli A, Maurizi G, Andreetti C, Ciccone AM, Ibrahim M, Piraino A (2016). Sleeve lobectomy versus standard lobectomy for lung cancer: functional and oncologic evaluation. Ann Thorac Surg.

[4] Ohata K, Zhang J, Ito S, Yoshimura T, Matsubara Y, Terada Y (2013). Right lower lobe sleeve resection: Bronchial flap to correct caliber disparity. Ann Thorac Surg.

[5] Boudaya MS, Abid W, Mlika M (2016). Sleeve right lower lobectomy: a rarely performed extended resection. Indian J Surg.

[6] Kocaturk CI, Saydam O, Sezen CB, Kalafat CE, Cansever L, Kutluk AC (2020). Is right sleeve lower lobectomy necessary? Is it safe?. Thorac Cardiovasc Surg.

[7] Wright CD (2006). Sleeve lobectomy in lung cancer. Semin Thorac Cardiovasc Surg.

[8] Waseda R, Iwasaki A (2018). Extended sleeve lobectomy: its place in surgical therapy for centrally located non-small cell lung cancer and a review of technical aspects. J Thorac Dis.

[9] Qu R, Ping W, Hao Z, Cai Y, Zhang N, Fu X (2020). Surgical outcomes of segmental bronchial sleeve resection in central non-small cell lung cancer. Thoracic Cancer.

